# *Piscine orthoreovirus* can infect and shed through the intestine in experimentally challenged Atlantic salmon (*Salmo salar* L.)

**DOI:** 10.1186/s13567-016-0343-z

**Published:** 2016-05-23

**Authors:** Helena Hauge, Maria Dahle, Torfinn Moldal, Even Thoen, Anne-Gerd Gjevre, Simon Weli, Marta Alarcón, Søren Grove

**Affiliations:** Norwegian Veterinary Institute, Pb 750, Sentrum, 0106 Oslo, Norway

## Abstract

*Piscine orthoreovirus* (PRV) is a ubiquitous virus in Norwegian salmon farms associated with the disease heart and skeletal muscle inflammation (HSMI). Experimental challenge has shown that the virus replicates in circulating red blood cells of Atlantic salmon prior to infecting heart myocytes. The infection route from water to blood is however still unknown. The related mammalian orthoreovirus primarily infects the lungs and gastrointestinal (GI) tract and is proposed to spread mainly through the faecal–oral route. To investigate the role of the salmonid GI tract in PRV-infection, oral and anal administration of virus was compared to intraperitoneal (i.p.) injection. When administered anally, PRV was transferred to blood 4 days post challenge (dpc) and levels peaked at 42 dpc, similar to i.p. injected fish. PRV was detected in heart and faeces with corresponding kinetics, and inflammatory heart lesions consistent with HSMI were observed from 49 dpc. The orally intubated group showed slower virus kinetics in both blood and heart, and no signs of HSMI. Compared to the oral and i.p. administration routes, leakage of virus inoculate by anal intubation was minor and challenge was restricted to the mid- and distal intestine. These findings show that anal intubation is an efficacious method for PRV delivery to the GI tract and demonstrates that PRV can establish infection through the intestine with the potential for transmission via faeces.

**Electronic supplementary material:**

The online version of this article (doi:10.1186/s13567-016-0343-z) contains supplementary material, which is available to authorized users.

## Introduction

In Norway, *Piscine orthoreovirus* (PRV) is associated with heart and skeletal muscle inflammation (HSMI) in Atlantic salmon (*Salmo salar* L.) [1–3]. HSMI is one of the most common diseases in Norwegian salmon farming [4] and is characterized by epicardial and myocardial inflammation, usually combined with inflammation in the red skeletal muscle and signs of circulatory disturbances [1]. The relationship between PRV infection and development of HSMI remains unclear as PRV infection is not always associated with disease. In Norway, HSMI typically occurs in seawater staged farmed Atlantic salmon although PRV is ubiquitous in seawater and freshwater salmon farms [5] and is occasionally detected in wild salmon [6, 7] and marine species [8]. PRV has also been commonly detected in salmon and trout species in Chile and western North America without the occurrence of HSMI [9–11]. Furthermore, despite replicating to high viral loads in Atlantic salmon, PRV from western North America failed to cause HSMI or other disease [12, 13]. Whether this is due to differential virulence of PRV or multifactorial disease triggers is not known.

PRV is a non-enveloped virus with a genome consisting of ten segments of double stranded RNA. The virus is related to the well characterized mammalian orthoreovirus (MRV) [14]. MRV primarily infects the lungs and GI tract and is so common in humans that most people are exposed early in life and develop immunity without any signs of disease [15]. Several isotypes of MRV have been characterized, and some are associated with gastroenteritis in young children. In murine models, MRV has also been found associated with pathology in the liver and heart [16]. Although the relative sequence homology is low, potential homologues of all main MRV protein-encoding genes have been detected in the PRV genome [17]. Studies of PRV by electron microscopy have so far revealed that the virus shape, size and viral factory formation in the cytoplasm of erythrocytes shares similarities to MRV [18]. Accordingly, it was of interest to determine the role of the GI tract in PRV infections.

**Figure 1:**
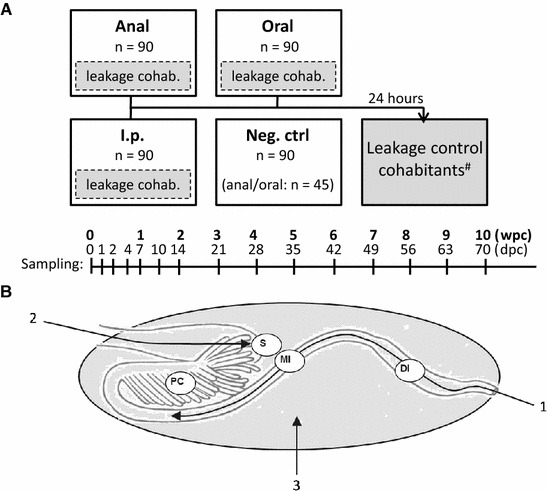
**Study design (A) and administration routes (B).** **A** Treatment groups (*n* = 90) were challenged as described in the “Materials and methods” section. To assess potential leakage of challenge inoculate, naïve fish were introduced to challenged groups after two rounds of water exchange, and then after 24 h transferred to a separate tank. Timeline specifies sampling schedule days post challenge (dpc) and weeks post challenge (wpc). ^#^Leakage control cohabitants were only sampled at 56 dpc. **B** Arrows indicate where challenge material was deposited and circles indicate where gastrointestinal samples were taken (stomach (S), pyloric caeca (PC), mid-intestine (MI) and the distal intestine (DI)). **B** modified with courtesy of Inge Bjerkås, Oslo, Norway.

The epithelial surfaces in e.g. gills, skin and intestine are major sites of viral entry in fish [19]. The intestine of Atlantic salmon posterior to the stomach has been defined into five different regions; the pyloric caeca, the first segment of the mid-intestine with pyloric caeca, the first segment of the mid-intestine posterior to pyloric caeca (in the following called mid-intestine), the second segment of the mid-intestine (in the following called distal intestine) and the posterior segment (Figure 1B) [20]. The distal intestine is characterized by a larger diameter than the mid-intestine and by pronounced complex folds in addition to simple folds. In contrast to mammals where mesenteric lymph nodes and distinct lymphoid follicles are harboured in the intestinal mucosa, teleost fish like Atlantic salmon have their immune competent cells including antigen presenting cells and T and B lymphocytes, more diffusely spread in the intestinal tissue [21]. Higher transcript levels of immune-related genes are observed in the distal intestine compared to the proximal regions [22], and the presence of cells that resemble mammalian M cells, important in stimulating mucosal immunity, has also been demonstrated in this segment [23].

PRV has been shown to transmit through cohabitation with infected fish, and controlled challenge trials have demonstrated that the virus infects and replicates in circulating red blood cells prior to infection of heart myocytes [18, 24, 25]. The infection route from water to blood is still unknown.

Studying the early events of infection presents obvious difficulties when using established experimental models such as i.p. injection and cohabitation. Ideally, fish should be challenged simultaneously with identical virus doses targeting the same infection site. Since MRV is detected in faeces and proposed to spread mainly through the faecal–oral route, we set out to study if a similar transmission route is relevant for PRV in Atlantic salmon, using an experimental model of oral and anal intubation of virus inoculates.

The current study shows that Atlantic salmon are efficiently infected by PRV after anal intubation, leading to transfer of virus from the intestinal lumen to blood, then heart and subsequently to development of HSMI. Furthermore, PRV was found to shed into faeces, indicating a potential virus transmission route. In contrast to established experimental models, the anal administration challenge route limits virus exposure mainly to the intestine and hence allows studies of viral infections over the epithelium at the very early time points.

## Materials and methods

### Fish and rearing conditions

The experimental study was carried out in accordance with the recommendations in the current animal welfare regulations: FOR-1996-01-15-23 (Norway), and the protocols were approved by the Norwegian Animal Research Authority. All fish handling was performed under anesthesia with Aqui-S vet. (Scan Aqua, Årnes, Norway) at a concentration of 10 mg/L, and the fish were monitored daily for mortality and signs of disease.

Unvaccinated Atlantic salmon (*Salmo salar* L.) pre-smolts (indigenous to the river Drammenselva, Buskerud County, Norway), 20–40 g (33.7 g average), originating from Hellefoss cultivation station, were transported to the research aquarium facility at the Norwegian Veterinary Institute. The fish were here reared in freshwater in standard 160 L fibreglass tanks with flow-through water supplied from the municipal water works. The water was passed through a carbon filter column and aerated mechanically in the aquarium facility, before entering the fish tanks. Water temperature and oxygen saturation was monitored daily and ranges were 8.3–12.1 °C and 86–99%, respectively, during the study period. The fish were hand-fed a 2 mm pelleted commercial diet (Skretting, Stavanger, Norway) at a rate of 2% of calculated biomass/tank/day. The fish were acclimatised for 3 weeks. Prior to the experimental start, histology was performed on 12 fish to rule out any health issues in the fish population, and three fish were tested for common salmonid viruses; PRV, salmonid alphavirus (SAV), piscine myocarditis virus (PMCV) and infectious pancreatic necrosis virus (IPNV) by real time RT-PCR using primers and probes as described elsewhere [7, 26–28].

### Preparation of virus homogenate

The PRV inoculum originated from a Norwegian field HSMI outbreak in 2012 and had previously been passaged through two controlled PRV challenge trials also resulting in HSMI where the challenge inoculum was prepared from homogenized PRV infected blood cells. Here, blood collected on heparinized tubes from PRV infected fish with HSMI was centrifuged, plasma removed, and the blood cell pellets stored frozen at −80 °C until use. At the day of infection, the pellets were diluted 1:2 in sterile phosphate-buffered saline (0.01 M, pH 7.4) (PBS) and combined, freeze-thawed in three rapid cycles until cell lysis, further diluted fivefold in PBS and used as challenge material. RNA corresponding to 25 µL of virus inoculate analysed by one-step real time RT-PCR targeting the PRV-segment λ1, resulted in a Ct value of 17. An inoculum dose of 0.1 mL, equivalent to Ct 15, was administered to the individual fish.

### Challenge experiment

Fish were kept in freshwater and starved for 6 days prior to challenge, partly to facilitate intubation and absorption of challenge material and partly to reduce potential leakage and rapid excretion of the inoculate e.g. via faeces. Treatment groups, comprising 90 fish each, were administered as follows under anesthesia with benzocaine (Figure 1):

*Anal intubation*: The challenge inoculate was administered by anal intubation using a sterile syringe fitted with a disposable mouse feeding needle (7202K, Fuchigami, Akirunoshi, Japan). After introduction via the anus, the feeding needle was inserted to a length where the 0.1 mL inoculate would become deposited in the mid-intestine (Figure 1B). Then the feeding needle was slowly withdrawn to minimize leakage.

*Oral intubation*: Fish were intubated using the same type of equipment and challenge material and the catheter was introduced through the mouth down to the stomach (bend) where challenge material was deposited (Figure 1B).

*Intraperitoneal injection (i.p.)*: Fish were injected i.p. with 0.1 mL inoculate.

*Negative controls*: Fish were intubated via the anal- or oral route respectively with 0.1 mL PBS.

*Leakage control*: In order to minimize adverse effects of inoculate leakage, i.e. infection via non-GI routes, the following procedures were undertaken. After challenge, fish were first dipped into a tank with fresh water and then left in a temporary tank with maximum water-flow (12 L/min) until all fish in the group had been treated. The water was then changed completely twice within the first 12 h before the fish were finally transferred to a permanent tank for the rest of the experiment. To further reduce potential excretion of infective material via intestinal waste, fish were not fed the first 24 h after intubation. To assess potential leakage of challenge material from challenged fish and the consequential risk of virus transmission via undesirable routes, 10 naïve fish (“leakage control cohabitants”) were introduced to the tanks of each of the three challenged groups after the two changes of water. These leakage control fish were left in the tank for 24 h, and then transferred to separate tanks where they were kept until 56 dpc when they were sacrificed and sampled.

Fish were monitored for a total period of 70 dpc where upon all remaining fish were sampled.

### Sampling

Six fish per group were euthanized with an overdose of Finquel vet. (Scan Aqua) and sampled at 15 individual time points after challenge, i.e. at 6 h post-challenge (in the following denoted 0.25 dpc) as well as 1, 2, 4, 7, 10, 14, 21, 28, 35, 42, 49, 56, 63 and 70 dpc.

Weight-, stomach- and intestinal content registration did not reveal any significant differences between experimental groups. Neither did haematocrit measurements.

### Detection of viral RNA

For virus analyses, samples of heart (tip), spleen, head kidney, stomach, pyloric caeca, mid- and distal intestine, and faeces were collected on RNAlater and stored at −80 °C until further processing. Total RNA was isolated manually (heart, stomach, faeces) or automatically (QIAsymphony, QIAGEN, Hilden, Germany) (pyloric caeca, mid-intestine, distal intestine, spleen, head kidney) with the RNeasy Mini Kit (QIAGEN) according to the manufacturer’s recommendations. 50 µL of blood was transferred to NucliSENS easyMag lysis buffer (280134, bioMérieux, Marcy l’Etoile, France) before isolation of nucleic acids (easyMag, bioMérieux). The RNA-concentration was measured using a NanoDrop™ 2000 spectrophotometer (Thermo Scientific, Wilmington, DE, USA), and the samples were stored in RNase-free water at −80 °C. Real time RT-PCR was performed on 500–1000 ng total RNA using OneStep RT-PCR kit (QIAGEN) and primers and probes targeting the PRV-segment λ1 as described elsewhere [7] and according to the manufacturer’s instructions. PCR was run for 45 cycles, and all virus negative samples were given an arbitrary Ct-value of 45 allowing them to be included in statistical analyses. Validation of assays and data handling was according to the MxPro Manual, and baseline and cycle threshold (CT) were set manually. Analyses were based on the mean Ct-value of six fish per group and time point.

### Histology

For histological examination, samples of gills, pseudobranch, skin, muscle, heart, liver, spleen, kidney, stomach, pyloric caeca, mid- and distal intestine were fixed in 10% buffered formalin. After 24–48 h of fixation, the samples were transferred to 70% ethanol until processed and embedded in paraffin wax. Samples from a total of 171 fish were sectioned, stained with haematoxylin & eosin (H&E) and examined by light microscopy as follows: all anally intubated fish, all negative controls until 63 dpc, oral group fish at 49 and 56 dpc, and i.p. group fish at 63 and 70 dpc. Throughout the study, histology showed that most of the fish examined suffered sparse gill inflammation. No consistent lesions were noted in muscle, pseudobranch, gastrointestinal tract, liver, kidney or spleen. A total of nine fish died; three in the anal group (14, 41 and 48 dpc), 1 (63 dpc) in the oral group, and 5 (2, 24, 24, 26, 28 dpc) in the control group. Mortalities displayed similar macroscopical and microscopical lesions; mild to moderate skin lesions related to *Saprolegnia*-infection and mild to moderate bacteria gill inflammation.

### Immunohistochemistry

A polyclonal antibody against the putative PRV outer capsid protein σ1 (anti-σ1) (kindly provided by Øystein Wessel, Norwegian University of Life Sciences, Oslo, Norway) was used to investigate the presence of viral proteins in hearts of anally infected fish. We used a previously described protocol for immunohistochemical detection of PRV with minor modifications [24]. In short, heart sections mounted on poly l-lysine coated glass slides were heated at 60 °C for 20 min, deparaffinized in xylene and rehydrated through graded alcohol baths. Antigen retrieval was done by microwave treatment for 2 × 6 min in citrate buffer (0.1 M, pH 6.0). To block non-specific binding sites, sections were incubated 20 min in goat serum diluted 1:50 in 5% skimmed dry milk in TBS (pH 7.6, 0.05 M Tris/HCl, 0.15 M NaCl). Primary antibody (anti-σ1) diluted 1:2500 in 1% skimmed milk in TBS was then added and the sections incubated in a humidity chamber at 4 °C overnight. The UltraVision ONE detection system kit (Thermo Scientific) was used for detection of bound antibody according to the manufacturer’s instructions, employing Fast Red (1 mg/mL) as substrate. Finally the sections were counterstained with haematoxylin and mounted (Aquamount, Thermo Scientific).

### Statistical analyses

Statistical analyses were performed using the JMP™ software (SAS Institute Inc., Cary, NC USA). Non-parametric Wilcoxon was used to compare groups and non-parametric Kendall’s τ and Spearman’s ρ was used for testing correlation.

## Results

### PRV levels in blood during the course of infection

**Figure 2:**
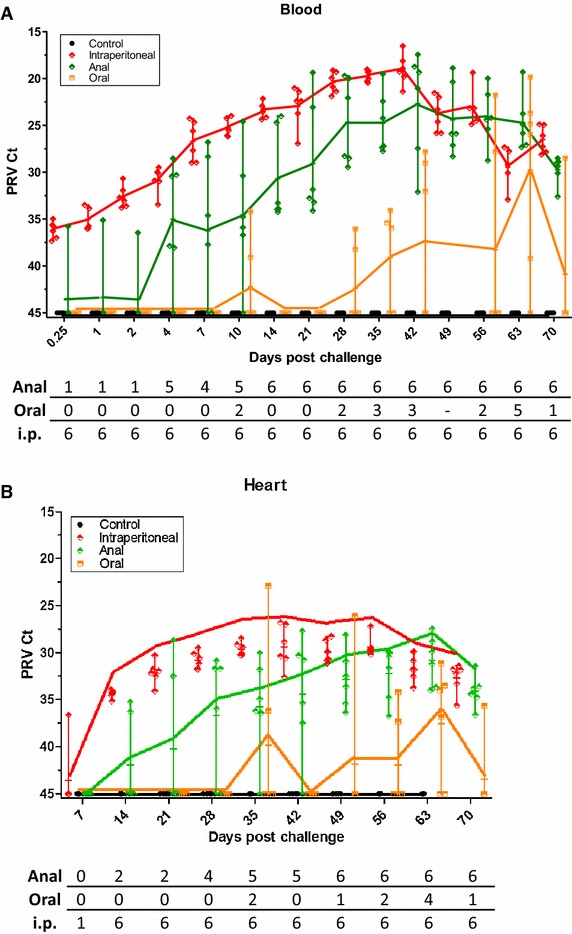
**PRV infection dynamics in blood and heart according to the different modes of challenge.** PRV levels (Ct values) in blood (**A**) and heart (**B**) of i.p. (red), oral (orange), anal (green) and control (black) group fish. Markers indicate individual fish, horizontal bars group means and vertical bars the range. Graph for control group and parts of graph for oral group has been slightly parallel offset from x = 45 for improved intelligibility. Table indicates number of positive fish per group (*n* = 6 for the challenge groups except for 70 dpc, *n* = 4 for control group).

PRV was detected by real time RT-PCR in blood samples in all the challenged groups but not in the control group (Figure 2A). In the i.p. group, PRV was detected in all sampled fish already 0.25 dpc, after which the mean level increased steadily until peaking at around 42 dpc (mean Ct 19). In the anally challenged group, only one single fish was positive at 0.25, 1 and 2 dpc, and then the number of positive fish increased until all fish were PRV positive from day 14. Peak levels were reached at 42 dpc (mean Ct 22.7), as observed for the i.p. injected fish. In contrast, PRV was only detected consistently in orally injected fish from 28 dpc and onward and even then never in all sampled fish. From 28 dpc, the mean level of PRV increased in orally challenged fish until 63 dpc when 5 out of 6 oral fish had PRV Ct values comparable to the other challenge groups.

### Temporal development in PRV levels in heart tissue

Also for heart tissue, PRV was detected by real time RT-PCR in all three challenged groups (Figure 2B). The data for heart were overall similar to the findings in blood, with the i.p. group exhibiting the earliest detection (one out of six fish at 7 dpc) and highest initial PRV levels. In the anal challenge group, the number of PRV positive fish increased from two out of six fish at 14 dpc until all fish were positive from 49 dpc and onward, with PRV-levels peaking at 63 dpc (mean Ct 30.8). The mean PRV level of the anal challenge group was accordingly significantly lower (*p* < 0.05) than in the i.p. group between 14 and 49 dpc, after which similar levels were reached. PRV was detected much later in the oral infection group and was never seen in every fish at any sampling point, peaking with four out of six fish positive at 63 dpc (mean Ct 37.6).

### Histology and PRV-immunohistochemistry of heart tissue

**Figure 3:**
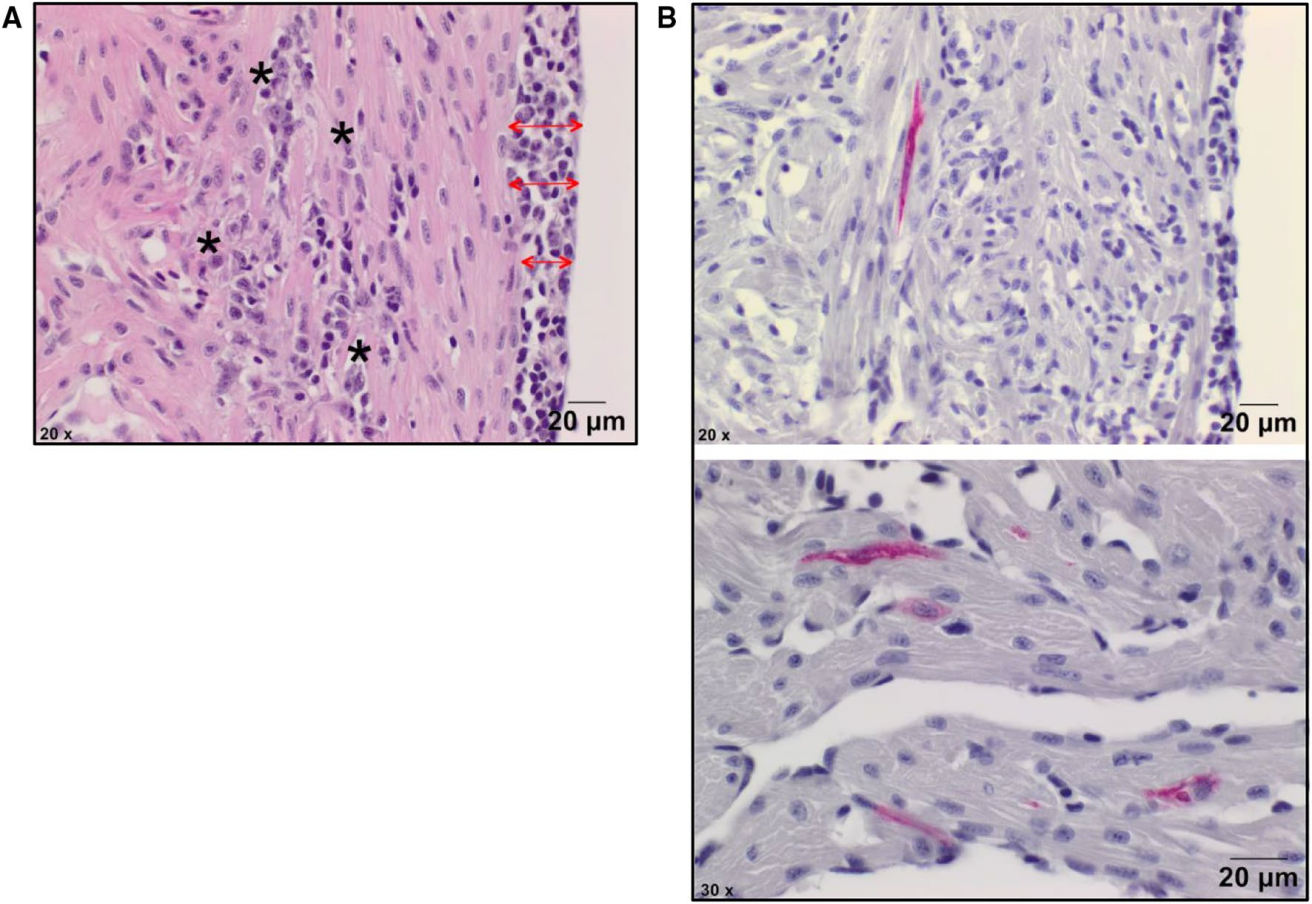
**Histology (A) and PRV immunohistochemistry (B) of hearts from anally intubated fish.** **A** Cardiac ventricle showing epicarditis (red arrows) and myocarditis in the underlying compact myocardium (asterisk) in the anal group at 9 wpc. **B** Immunostaining of heart tissue in the anal group at 9 wpc. PRV antigen detected with σ1 antibodies in cardiomyocytes in the cardiac ventricle (red colour).

Inflammatory heart lesions consistent with HSMI were observed in 5 out of 12 fish in the i.p. group (63–70 dpc) and 4 out of 24 fish in the anal group (49–63 dpc) (Figure 3A and Table 1). Minor myocarditis was seen at early samplings in a few individuals. The fish in the oral and control groups showed no HSMI lesions.

**Table 1:** **Histology and immunohistochemistry for heart tissue**

wpc dpc	0–4 0.25–28	5 35	6 42	7 49	8 56	9 63	10 70
Anal (IHCM)	0/54 (0/10)	0/6 (1/2)	0/6 (2/2)	1/6 (2/6)	1/6 (2/6)	2/6 (1/1)	0/6 (0/1)
Oral (IHCM)	0/6	–	–	0/1 (0/1)	0/2 (0/2)	–	–
i.p.	–	–	–	–	–	4/6	1/6
Neg. ctrl.	0/26	0/2	0/3	0/3	0/3	0/3	–

Number of fish with HSMI in the anal, oral, i.p. and negative control group at different time points. Positive fish are expressed out of the total analysed fish per group. In parentheses the number of positive fish detected in situ by immunohistochemistry.

Hearts from fish with high PRV-levels in blood (Ct < 29) in the anally intubated group were further analysed by immunohistochemistry, and positive staining was detected in 8 out of 28 analysed fish (35–63 dpc) (Table 1). At 35 and 42 dpc, PRV positive cells were mainly circulating blood cells located in the cardiac lumen (suspected erythrocytes and leukocyte-like cells). At later time points PRV was found mainly in cardiomyocytes (Figure 3B). Positive staining was not observed at earlier (0.25–28 dpc) or at the last sampling point (70 dpc).

### Distribution dynamics of PRV in gastrointestinal tract of challenged fish

**Figure 4:**
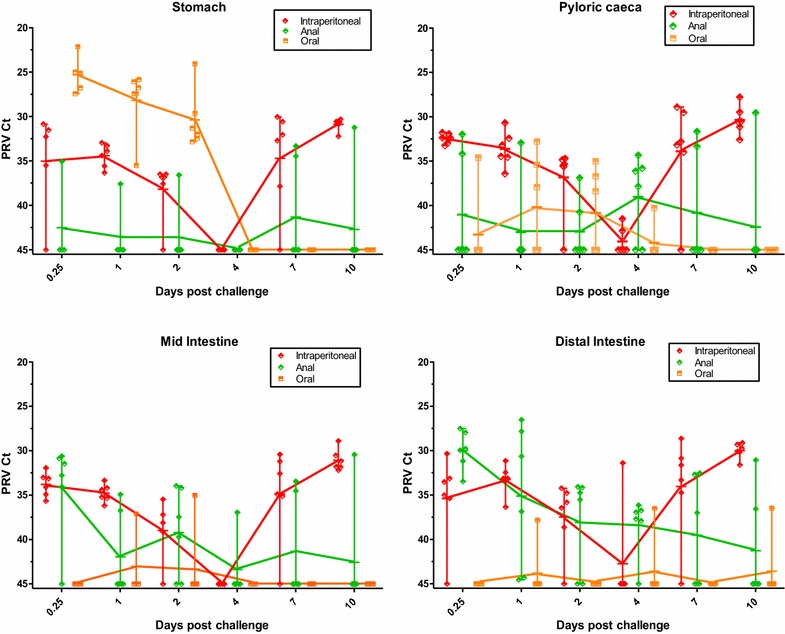
**Distribution dynamics of PRV in the GI tract of challenged fish.** Viral loads (Ct values) in four segments of the gastrointestinal tract from 0.25 to 10 dpc by i.p. (red), oral (orange), anal (green) administration routes. Markers indicate individual fish, horizontal bars group means and vertical bars the range. Graph for control group and parts of graph for oral group has been slightly parallel offset from x = 45 for improved intelligibility.

To investigate the distribution of PRV during the early phase post challenge, four segments of the GI tract, from the stomach to the distal intestine, (Figure 1B) were analysed by PRV real time RT-PCR from 0.25 to 10 dpc (Figure 4). The data showed that both tissue distribution and distribution dynamics differed between the challenge groups in accordance with the different administration routes. For the i.p. group, PRV levels were comparable between the four GI segments at all sampling times, indicating a uniform distribution of virus in the peritoneum. From a medium high level (mean Ct 32.4–35.4) at 0.25 dpc, the PRV levels gradually decreased until a distinct minimum was reached by 4 dpc, after which the PRV levels increased in all four segments, in line with virus replication in blood. Compared to the i.p. group, the anal and oral challenge groups exhibited clear differences in the distribution of virus in the four GI segments. At early time points, PRV levels in the oral challenge group were distinctly higher in stomach where the inoculum was deposited, compared to the other segments. From high levels in stomach at 0.25 dpc, PRV amounts gradually decreased during 1 and 2 dpc until becoming undetectable at 4 dpc. In pyloric caeca, three of six oral fish were slightly PRV positive (Ct > 40) at both 1 and 2 dpc, after which PRV became undetectable at 7 dpc. In the mid- and distal intestine, PRV was only detected at low levels in 5 out of 36 fish from 0.25 to 10 dpc (Ct > 35). In the anal challenge group, PRV levels in stomach and pyloric caeca were consistently low with only a few detections, except for in pyloric caeca at 4 dpc when 4 out of 6 fish were positive (Ct > 34). In line with the deposition of the virus inoculum, PRV was detected in mid- and distal intestine at relatively high levels at 0.25 dpc (mean Ct 34.1 and 30.0, respectively), gradually decreasing until 10 dpc when PRV was still detectable in 2 out of 6 fish. In contrast to the i.p. and oral challenge groups, the anal challenge group did not exhibit the same marked minimum in PRV detection at 4 dpc.

### PRV in faecal contents

**Figure 5:**
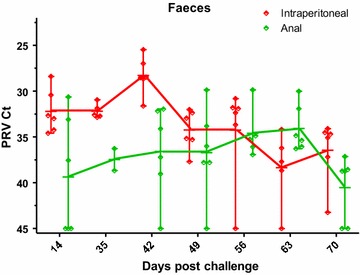
**PRV in faecal contents.** PRV levels (Ct values) in faeces samples from i.p. (red) anal (green) groups. Markers indicate individual fish, horizontal bars group means and vertical bars the range.

Since inoculated virus was detectable in the intestine of anally challenged fish as late as 10 dpc, only faeces samples collected from the intestine of the i.p. and anal group fish from 14 dpc and later were analysed for viral RNA. PRV was detected in faeces of both groups, reaching a maximum at 42 dpc (mean Ct 28.3) in the i.p. fish and at 63 dpc (mean Ct 34.1) in the anal challenge group, corresponding to the virus levels in blood (Figure 5).

### Leakage of PRV from challenged fish

**Figure 6:**
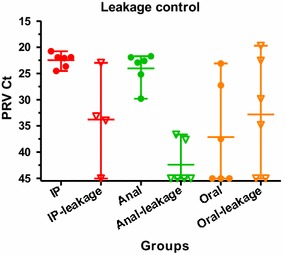
**Viral loads (Ct-values) in leakage control cohabitants and challenge groups at 56 dpc.** Pooled samples of heart, spleen and head kidney tissue from leakage groups (i.p.-leakage, anal-leakage and oral-leakage) were analysed by PRV qPCR and compared to the corresponding samples from challenge groups (i.p. anal and oral). Markers indicate individual fish, horizontal bars group means and vertical bars the range.

To assess potential leakage of inoculated virus from challenged fish, PRV real time RT-PCR was applied on samples of pooled heart-, spleen- and head kidney tissue from leakage control cohabitants (leakage) at 56 dpc and compared to the corresponding samples from challenged fish. The results demonstrated notable differences between the leakage groups (Figure 6). While most fish were positive for PRV in the oral leakage group and quite surprisingly also in the i.p. leakage group, the majority of fish in the anal leakage group were negative. The mean PRV Ct values in the oral leakage group was significantly lower (*p* < 0.01) than that of the anal leakage group, indicating a significantly higher viral load in the former group. Between the i.p. leakage and anal leakage groups, a similar difference was apparent but not significant (*p* = 0.09).

### Integrated analyses

To further characterize the kinetics of the PRV-infection, PRV levels in GI segments, heart, faeces and blood were analysed for possible correlations in the i.p. and anal groups. Comparable levels of correlation were observed for anal and i.p. challenge groups but due to a generally lower variation in Ct values in the i.p., only data from this group are presented in Additional files 1, 2 and 3. Comparison of PRV levels in the four GI segments versus blood from 4 to 14 dpc, showed a strongly significant correlation for all four GI tissues (*p* < 0.0001 by non-parametric Spearman’s ρ testing) (Additional file 1). A strongly significant correlation was also found between PRV levels in heart and blood (*p* < 0.0001 by non-parametric Spearman’s ρ testing). When sampling time was taken into account, it became clear that a certain spatiotemporal development took place during the infection (Additional file 2). For all sampling time points, the PRV Ct values for blood and heart grouped into relative narrow clusters in the “blood-heart space”. The centre of these clusters further exhibited an interesting development with time. Between 7 and 21 dpc, heart seemed to have a comparatively stronger increase in PRV levels which was then succeeded by a more equal increase in both tissues until the peak was reached at 42 dpc. After 42 dpc, PRV levels decreased slowly in heart but more rapidly in blood. PRV levels in blood and faeces were strongly correlated (*p* < 0.0001 by non-parametric Spearman’s ρ testing) and again the PRV Ct values in these samples formed sampling time-dependent clusters (Additional file 3). In contrast to blood-heart, the temporal development of these blood-faeces clusters followed a relative linear trajectory, in particular after 42 dpc where both sample types experienced a comparable decrease in PRV levels.

## Discussion

Understanding how pathogenic organisms enter their host and spread in the environment is crucial for the management of disease. For PRV, the mechanisms of infection and transmission are yet unknown. In this study, we investigated if the GI tract could serve as a putative site of infection and potential source of shedding for PRV as shown for the related mammalian orthoreovirus [15].

A cohabitation challenge model is often preferred in viral infection studies because the transfer of virus to the cohabitants mimics natural infection. However, in order to study site-specific virus–host interactions at the first encounter it is essential to control when and where the challenge takes place, as well as standardizing the viral dose. Whereas injecting virus into the peritoneum of the host is assumed to ensure these factors, the intrinsic defense mechanisms of the mucus, skin, gills and gut of the fish are bypassed, and the infection cannot be considered natural. We therefore set out to establish an experimental model for studying PRV-infection with particular focus on the GI tract at early time points post infection. The GI tract is responsible for uptake of water and nutrients but also serves as a protective barrier towards the environment. Intestinal challenge may therefore mimic the conditions of a natural infection. The intestinal epithelium is very likely to be an entry port for pathogens transmitting through the fecal-oral route, which include orthoreoviruses in mammals and possibly also PRV. In this study, a PRV inoculum was administered to fish via either oral or anal intubation or by i.p. injection, the latter serving as a basis for comparison. Analysis of the PRV levels in blood and heart showed that anal, but not oral intubation of virus resulted in infection kinetics comparable to i.p. injected fish. Histology further demonstrated that fish infected through the anal route develop HSMI. The real time RT-PCR analysis of GI samples showed that the challenge inoculum is largely restricted to stomach and pyloric caeca in the orally intubated group and to the mid- and distal intestine in the anally intubated group. The low levels of virus RNA in mid- and distal intestine of orally challenged fish suggest that only limited amounts of virus reached this compartment, which could be due to the apparent leakage of virus to the water column that was likely caused by regurgitation. The relative delayed and milder infection kinetics of the oral group may hence correspond to a lesser amount of infective material reaching the intestinal segments that likely serve as an infection site for PRV. Oral and anal vaccination against enteric redmouth disease in rainbow trout revealed that much of the orally fed antigen is digested in the stomach before it reaches the second segment of the intestine where it can be taken up as an immunogenic antigen [29], and this may also in part provide an explanation for the reduced PRV load observed in our oral treatment group.

A feature of mammalian reovirus is that it depends on proteolytic activity for optimal infection. Specific proteolysis of the outer capsid to form an infective subviral particle (ISVP) triggers uptake of the virus, and endosomal proteolysis and low pH further induces virus transfer into the cell cytoplasm [30]. Analysis of the PRV µ1 protein sequence has revealed the presence of a central proteolytic cleavage site, indicating that similar mechanisms may apply for PRV [17]. MRV given orally is demonstrated to be readily transferred to the intestine (within 10–30 min) as ISVPs, most likely due to intestinal protease activity and acidic conditions, indicating that oral uptake should be well suited for reoviruses [31]. Chymotrypsin activity (<10 µg/mL) can trigger MRV ISPV formation and increase infectivity ex vivo, but high levels (>100 µg/mL) may have an inhibitory effect [32]. Compared to mammals, the salmonid stomach is less acidic [33], and starvation has been demonstrated to increase the chymotrypsin levels in the pyloric caeca [34], factors that may affect PRV infectivity. In contrast to PRV, a recent report on IPNV reported slightly higher antigen uptake by oral than anal administration [35]. IPNV has been shown to resist harsh environments well [36, 37] but as PRV is also a non-enveloped virus, this difference may reflect separate infection strategies. It should also be noted that fish in the IPNV-study were starved only 24 h prior to intubation as compared to 6 days in the present study that in our experience was necessary to empty the intestine and enable challenge of the mid-intestine. Under natural circumstances, it is more likely that virus is introduced orally than anally. It could still be that infective virus is able to pass through the stomach and reach the intestine if embedded in fat-containing biological material, i.e. faeces or feed, which could protect from acids and digestive enzymes in the stomach. If the trial period had been extended or the virus inoculum absorbed to a matrix, results might have shown that a PRV-infection can be established also through the oral route.

To control for infection via non-GI routes, naïve fish were cohabitated with challenged fish for 24 h and then held in the same unit until 56 dpc. Although this could allow for individual infected fish to shed virus and further infect the cohort, analysis revealed interesting differences between the various challenge groups. While the relative higher virus levels in the oral leakage group could be explained by leakage of inoculate through regurgitation, the data suggest that the inoculate delivered anally remain relatively confined at the site of inoculation, which is somewhat surprising given the quite direct contact with the water through the anal opening. Although not statistically significant, results indicate that virus can leak through the narrow puncture in the peritoneal wall of i.p. injected fish. In cohabitation challenge experiments, commonly performed with 30–50% of i.p. injected shedders, cohabitant infection efficacies often vary and may be influenced by virus leakage to water from the i.p. injected fish. Moreover, the i.p. challenge method is considered particularly targeted, but leakage short-circuiting the intended i.p. route could add a potentially significant mucosal/immersion component to this challenge type. In comparison, the low leakage observed in the anal challenge, suggest this route to be relatively targeted.

Regarding the kinetics of the PRV-infection, the observed correlation between PRV levels in blood and heart is complex, illustrated by the non-straightforward spatiotemporal development (Additional file 2). These data suggest that heart has an “eigenvalue” in the pathogenesis of PRV in which heart tissue contributes independently to overall virus load, e.g. by local replication. In contrast the relation between PRV in blood and faeces is simpler, almost linear (Additional file 3). This suggests that PRV levels in faeces can be explained by blood levels alone, and that intestine hence is less likely to contribute to faeces levels by local replication. The strong correlation between PRV levels in blood and the four GI segments (Additional file 1) further corroborates the determining role of blood for the PRV findings. Hence, the demonstration of relatively high levels of PRV RNA in faeces samples suggests that faeces is a likely route for transmission of PRV, and that PRV reaches the intestinal lumen via active/passive transport from blood across the intestinal wall. A study on SAV similarly showed that virus was detected in faeces and mucus following challenge [38]. These observations suggest that analysis of fish droppings collected from the net pens or freshwater tanks could be used as a diagnostic tool for early detection of virus without sacrificing fish.

In conclusion, this study demonstrates that anal intubation is an efficacious and well suited model for studying PRV-infection. Furthermore, the results show that PRV can establish an infection through the intestine and can likely shed to the environment through faecal contents. To our knowledge, this is the first report to document that the intestine can be a port of entry for PRV under experimental conditions. The more specific PRV infection mechanisms should be a subject for future studies.

## Additional files

10.1186/s13567-016-0343-z **Correlation between PRV levels (Ct values) in blood and four gastrointestinal tissues of i.p. fish.** Markers indicate individual fish sampled at 4 (filled circle), 7 (square), 10 (star) and 14 (triangle) dpc. Note the inverted axes.
10.1186/s13567-016-0343-z **Temporal development in relationship between PRV levels in blood and heart of i.p. group from 7 dpc to 70 dpc.** Fish from each sampling time point are marked according to legend insert *Day*-*Group*. Coloured circles indicate the approximate centre of distribution of PRV values for each sampling time point as indicated by inserted coloured text boxes. The arrows indicate potential temporal trends in PRV distribution centre in the course of the experiment.
10.1186/s13567-016-0343-z **Temporal development in relationship between PRV levels in blood and faeces of i.p. group from 7 dpc to 70 dpc.** Fish from each sampling time point are marked according to legend insert *Day*-*Group*. Coloured circles indicate the approximate centre of distribution of PRV values for each sampling time point as indicated by inserted coloured text boxes. The arrows indicate potential temporal trends in PRV distribution centre in the course of the experiment.

## Abbreviations

dpc: days post challenge
GI: gastrointestinal
HSMI: heart and skeletal muscle inflammation
IPNV: infectious pancreatic necrosis virus
i.p.: intraperitoneal
MRV: mammalian orthoreovirus
PMCV: piscine myocarditis virus
PRV: *Piscine orthoreovirus*
SAV: salmonid alphavirus
