# Supplementary figures and images for: Piscine orthoreovirus can infect and shed through the intestine in experimentally challenged Atlantic salmon (Salmo salar L.)
Source: Vet Res. 2016 May 23;47:57. doi: 10.1186/s13567-016-0343-z (PMC4877738; doi:10.1186/s13567-016-0343-z)

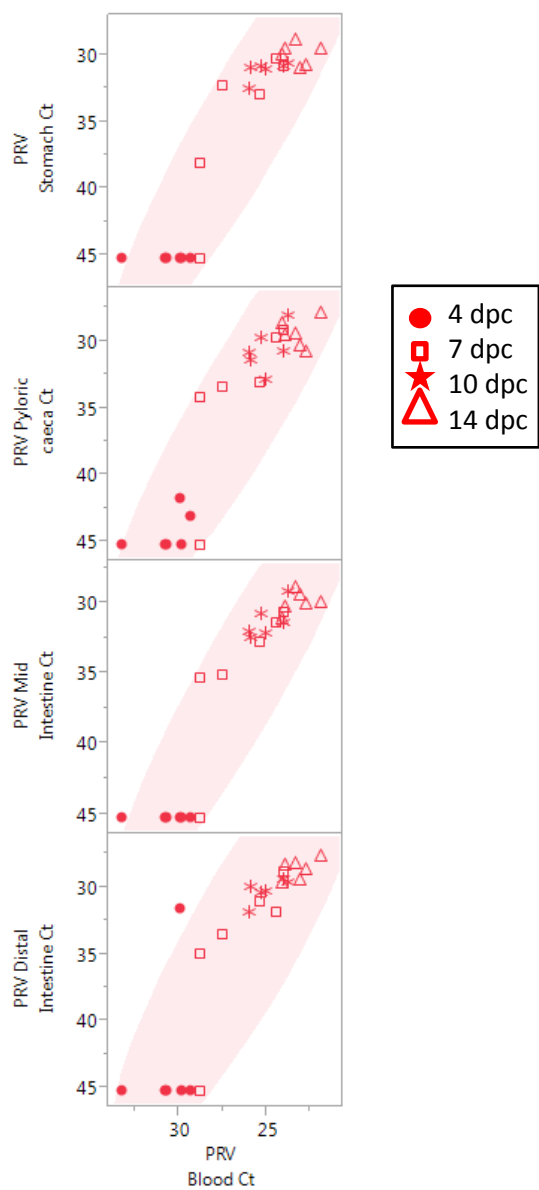

Supplement: Supplementary file 1 — 10.1186/s13567-016-0343-z Correlation between PRV levels (Ct values) in blood and four gastrointestinal tissues of i.p. fish. Markers indicate individual fish sampled at 4 (filled circle), 7 (square), 10 (star) and 14 (triangle) dpc. Note the inverted axes. [file 13567_2016_343_MOESM1_ESM.pdf]

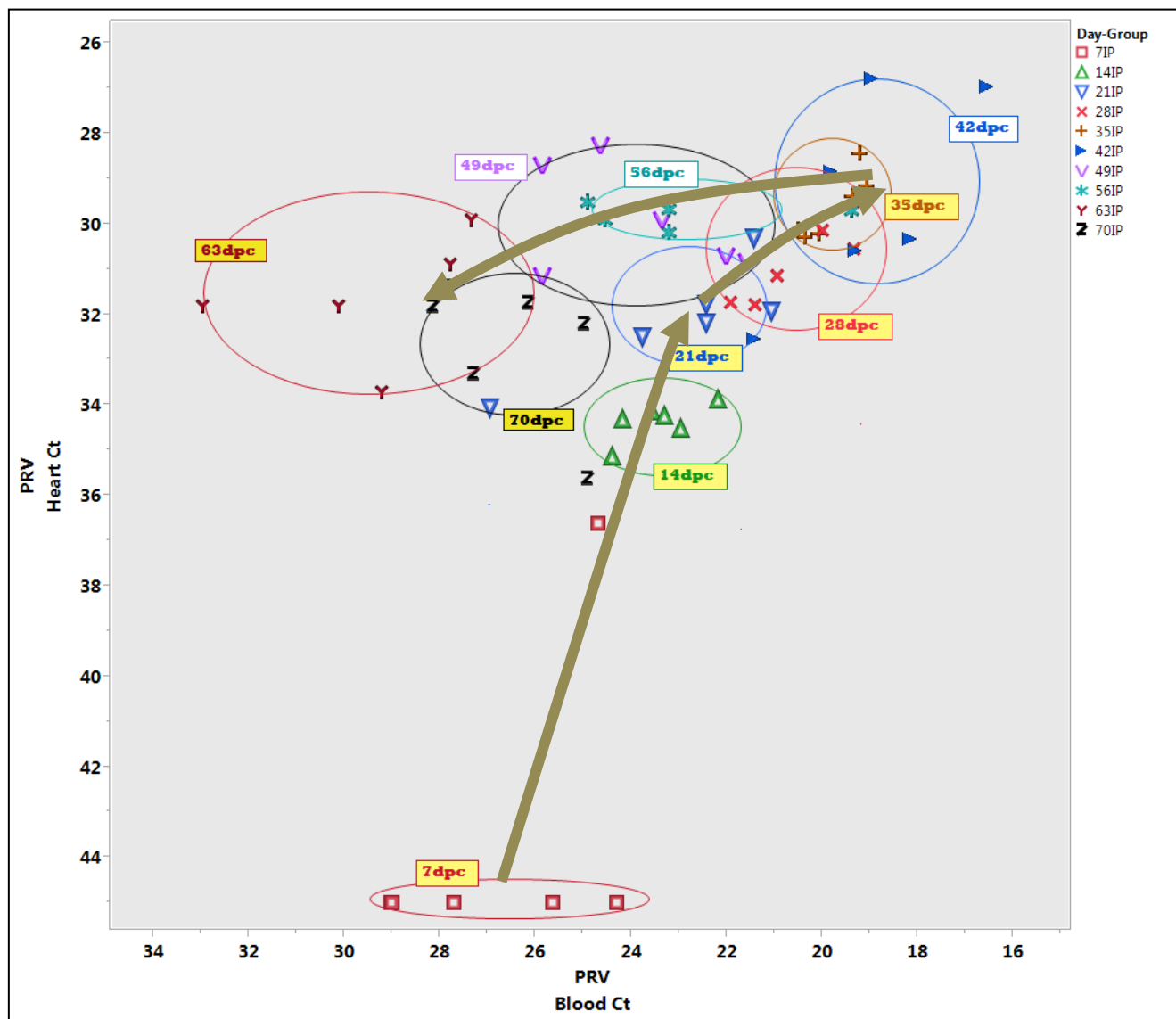

Supplement: Supplementary file 2 — 10.1186/s13567-016-0343-z Temporal development in relationship between PRV levels in blood and heart of i.p. group from 7 dpc to 70 dpc. Fish from each sampling time point are marked according to legend insert Day-Group. Coloured circles indicate the approximate centre of distribution of PRV values for each sampling time point as indicated by inserted coloured text boxes. The arrows indicate potential temporal trends in PRV distribution centre in the course of the experiment. [file 13567_2016_343_MOESM2_ESM.pdf]

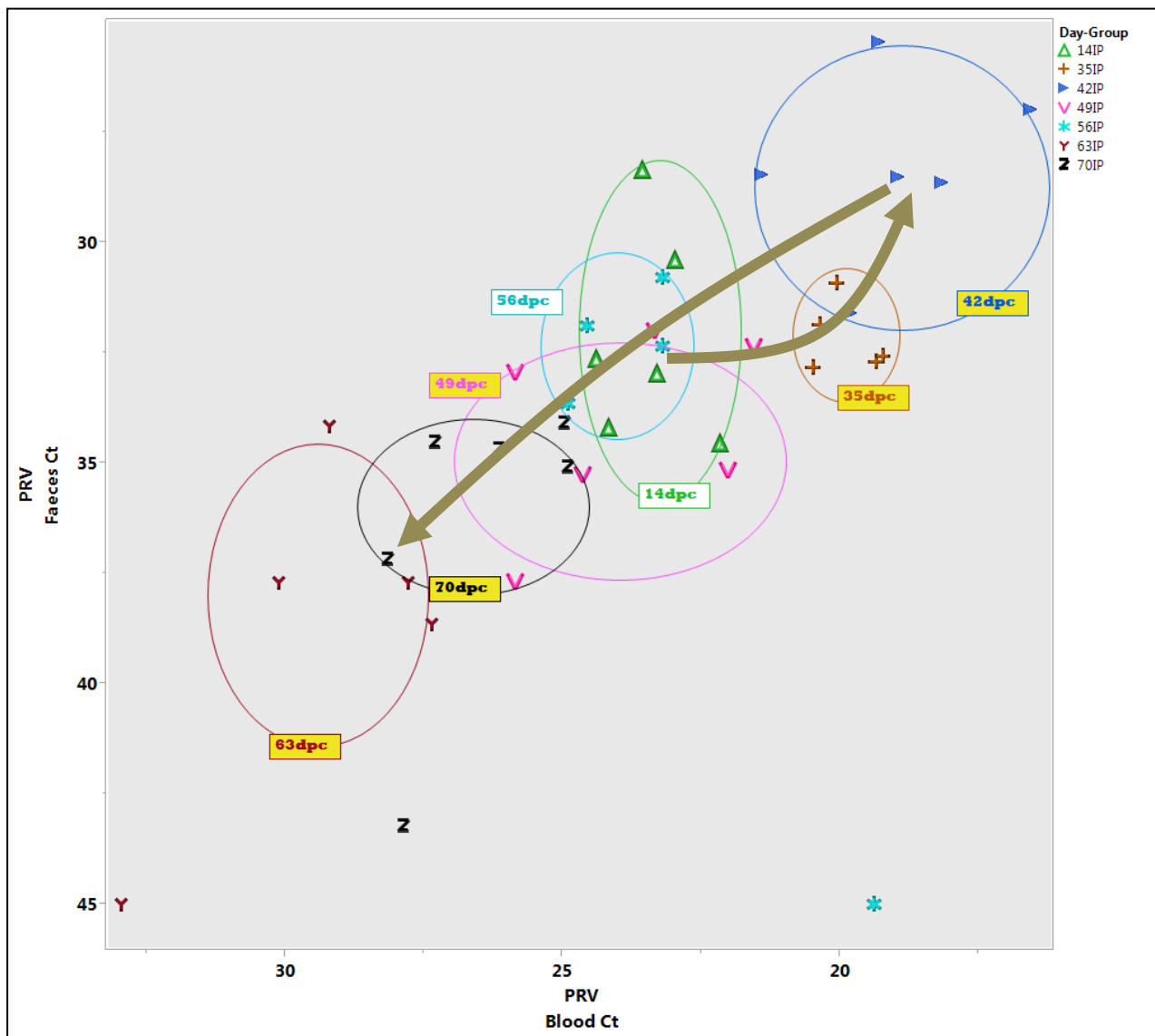

Supplement: Supplementary file 3 — 10.1186/s13567-016-0343-z Temporal development in relationship between PRV levels in blood and faeces of i.p. group from 7 dpc to 70 dpc. Fish from each sampling time point are marked according to legend insert Day-Group. Coloured circles indicate the approximate centre of distribution of PRV values for each sampling time point as indicated by inserted coloured text boxes. The arrows indicate potential temporal trends in PRV distribution centre in the course of the experiment. [file 13567_2016_343_MOESM3_ESM.pdf]
